# Fighting vessel dysmorphia to improve glioma chemotherapy

**DOI:** 10.15252/emmm.201708431

**Published:** 2017-11-10

**Authors:** Marja Lohela, Kari Alitalo

**Affiliations:** ^1^ Biomedicum Imaging Unit University of Helsinki Helsinki Finland; ^2^ Translational Cancer Biology Program University of Helsinki Helsinki Finland; ^3^ Wihuri Research Institute University of Helsinki Helsinki Finland

**Keywords:** Cancer, Neuroscience, Vascular Biology & Angiogenesis

## Abstract

High‐grade gliomas are aggressive and abundantly vascular tumors, and as in most cancer types, blood vessels in advanced lesions are highly abnormal. Poor perfusion and vascular leakage in tumor tissue resulting in hypoxia, necrosis, and high interstitial fluid pressure can hamper the efficient delivery of chemotherapy. Tumor angiogenesis is known to be supported by host leukocytes recruited to the tumor microenvironment, but the mechanisms leading to dysfunctional vascular network formation are incompletely understood. In this issue of *EMBO Molecular Medicine*, Mathivet *et al* (2017) present an elegant study, where longitudinal intravital imaging gives new insight on how recruitment and polarization of tumor‐associated macrophages regulate aberrant angiogenesis in experimental gliomas. They show that macrophage targeting results in vessel normalization and improved chemotherapy response, suggesting that the combination of these therapeutic modalities could improve the outcome of glioma treatment in the clinic.

The vasculature in solid tumors is almost always highly abnormal in both structure and function, with a tortuous, disorganized and even discontinuous architecture, uneven diameters, and leakiness arising from features such as abnormal endothelial junctions, incomplete pericyte coverage, and altered structural components. As they grow, tumors become poorly perfused and therefore hypoxic, and develop a high interstitial pressure. Metastasis may furthermore be facilitated by the compromised endothelial integrity (Carmeliet & Jain, 2011). Anti‐angiogenic therapeutic agents induce pruning of the excessive and abnormal vascular network, making the remaining vessels more stable and functional; this phenomenon is known as vascular normalization. The perfusion and oxygenation of tumors is thus improved, and this may limit invasion and metastasis as well as improve chemotherapeutic drug access to the tumor. High‐grade gliomas usually present an extreme case of tumor angiogenesis, but anti‐angiogenic therapy has so far not lived up to expectations in this tumor type (Wang *et al*, 2017).

Different types of host cells in the tumor microenvironment, notably cancer‐associated fibroblasts and various myeloid cells, stimulate angiogenesis and release angiogenic growth factors (Hanahan & Coussens, 2012). Gliomas release cytokines to recruit tumor‐associated macrophages (TAMs), which in the case of brain tumors arise from bone marrow‐derived monocytes or from yolk sac‐derived microglia (Quail & Joyce, 2017). Tumor‐associated macrophages in turn supply pro‐tumorigenic factors. Large numbers of TAMs and high levels of the macrophage mitogen colony‐stimulating factor (CSF)‐1 correlate with poor prognosis in most human tumors (Mantovani *et al*, 2017). Genetic or therapeutic targeting of the CSF‐1/CSF‐1 receptor (CSF‐1R) pathway in different tumor models leads to varying degrees of macrophage depletion; this has been found to have direct anti‐tumor activity or to potentiate the response to chemotherapy, often with anti‐angiogenic effects (Mantovani *et al*, 2017; Lohela *et al*, 2014). Depletion of macrophages reduced tumor growth also in some glioma models (Quail & Joyce, 2017; Zhai *et al*, 2011), and interestingly, small molecule inhibition of CSF‐1R was found to dramatically block tumor growth and progression by downregulating markers of M2‐like polarization of TAMs in a proneural model of glioblastoma (Pyonteck *et al*, 2013). However, in another glioma model, myeloid cell depletion led to accelerated tumor growth (Galarneau *et al*, 2007).

Intravital microscopy has emerged as an indispensable tool in studying the complex tumor microenvironment *in situ*, with the possibility of following in real time the dynamic interactions between cancer cells, immune cells, vasculature, and the extracellular matrix, and of following tumor growth and invasion longitudinally via imaging windows. In the study by Mathivet *et al* (2017; see Fig 1 for a graphical summary of the findings), gliomas arising from intracranially injected spheroids were first imaged by two‐photon microscopy through a cranial window at 2 weeks postimplantation. The authors found that at this stage, angiogenesis was proceeding in an orderly fashion, with tip cell‐guided sprouting and without the characteristics of aberrant angiogenesis normally observed in tumors. However, at 5 weeks, the same tumors displayed abnormal filopodia extension and sprouting along the stalk of the vessels, and significantly increased vessel diameters. Searching for the reason for the switch in vascular phenotype during tumor progression, the authors noticed that bone marrow‐derived macrophages accumulate in the tumors during this period, becoming increasingly concentrated around the tumor blood vessels. Further analysis showed that also the phenotype of the TAMs changed: At 2 weeks, their expression profile resembled that of classically activated, M1‐polarized macrophages. At 5 weeks, most of the TAMs were clustered in perivascular locations and had the alternatively activated M2 phenotype, which is known to include the VEGF‐secreting, highly angiogenic Tie2‐expressing subpopulation (Mantovani *et al*, 2017). Indeed, the M2‐type macrophages in late‐stage gliomas expressed VEGF in the immediate vicinity of endothelial cells. The accumulation of M2 macrophages coincided with an increasingly abnormal vessel structure also in human glioma samples.

**Figure 1:**
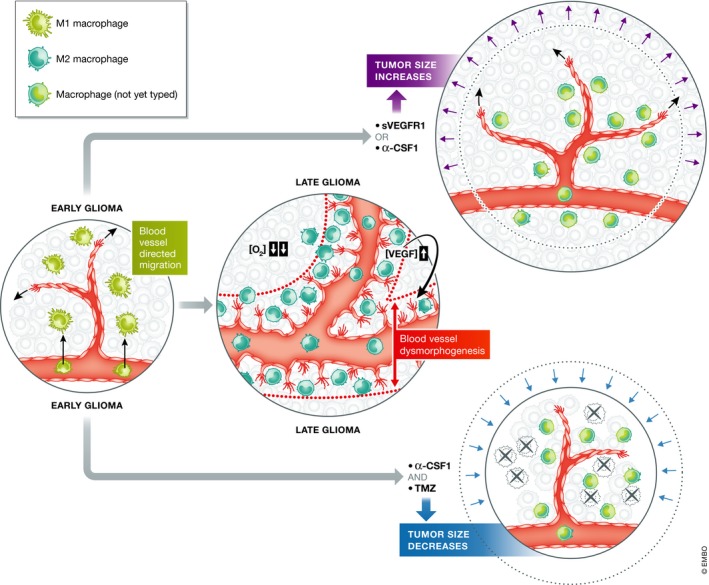
Macrophage depletion treatment by targeting CSF‐1, in combination with temozolomide chemotherapy, leads to increased glioma cell death (and improved outcome) in mouse xenografts Initially mainly M1 macrophages populate the tumor stroma, and vessels undergo sprouting angiogenesis. Upon tumor progression, M1 to M2 macrophage switch, perivascular recruitment, and increased production of VEGF occur, and tumor vessels expand, showing vascular dysmorphogenesis. Neutralization of VEGF by using the soluble extracellular domain of VEGFR1, or depletion of about half of the macrophages by using CSF‐1 blocking antibodies, alleviates hypoxia and normalizes tumor vessels, but tumor size increases. A combination of the CSF‐1 neutralizing antibody and the drug of choice, temozolomide, leads to increased apoptosis of glioma cells and improved survival of the mice.

Mathivet and coworkers then employed an anti‐CSF‐1 antibody to target the TAMs in gliomas (Mathivet *et al*, 2017). The therapy reduced TAMs by 50% and resulted in a dramatic normalization of the vascular network. Genetic depletion of VEGF from myeloid cells or administration of soluble VEGFR1 had the same effect, despite the presence of normal numbers of TAMs in the tumors, indicating that macrophage‐derived VEGF is the main culprit for vessel dysmorphia in this model. Strikingly, both TAM depletion and macrophage‐specific VEGF deletion accelerated tumor growth significantly, and conversely, treatment with recombinant CSF‐1 delayed glioma growth while accelerating the vessel dysmorphia. The vascular normalization caused by macrophage targeting presumably allowed better delivery of oxygen and nutrients to the tumor. Importantly, however, anti‐CSF‐1 therapy also seemed to improve the access of the standard‐of‐care chemotherapy temozolomide to gliomas, with a significant increase in median survival when compared to chemotherapy alone. This finding is congruent with findings in mammary tumors, where myeloid cell‐specific deletion of VEGF similarly led to vascular normalization and accelerated tumor growth, but improved the chemotherapy response (Stockmann *et al*, 2008). It was not reported by Mathivet *et al* (2017) if the anti‐CSF‐1 treatment also changed the phenotype of the TAMs that were not depleted, perhaps downregulating M2‐type effectors, as shown for the small molecule CSFR‐1 inhibitor in the proneural glioblastoma model (Pyonteck *et al*, 2013).

A recent meta‐analysis of clinical trials in high‐grade gliomas shows that while there is an overall increase in progress‐free survival with anti‐angiogenic therapy, overall survival is not significantly increased; however, there is a trend toward better outcome for combinations with chemotherapy when compared to anti‐angiogenic agents alone (Khasraw *et al*, 2014; Wang *et al*, 2017). Macrophage‐targeted therapies may offer an avenue to disable the tumor‐supporting microenvironment more profoundly than specific anti‐vascular strategies. Several clinical trials are currently ongoing with small molecule inhibitors or the more specific monoclonal antibodies against CSF‐1R (Peyraud *et al*, 2017). Monotherapy of solid tumors, including recurrent glioblastoma multiforme, has so far given disappointing results (Peyraud *et al*, 2017), and the findings of Mathivet *et al* (2017) may offer some explanation for this. Trials where anti‐CSF‐1R agents are combined with chemotherapy, immunotherapy, or targeted therapy are still ongoing, with only some very preliminary promising results (Peyraud *et al*, 2017), but fighting on several fronts could be the way forward in the treatment for aggressive tumors such as high‐grade gliomas.
